# On the role of TFEC in reptilian coloration

**DOI:** 10.3389/fcell.2024.1358828

**Published:** 2024-02-07

**Authors:** Athanasia C. Tzika

**Affiliations:** Laboratory of Artificial and Natural Evolution (LANE), Department of Genetics and Evolution, University of Geneva, Geneva, Switzerland

**Keywords:** reptiles, coloration, skin, chromatophores, ball python, snakes, TFEC, anole lizard

## Abstract

Reptilian species, particularly snakes and lizards, are emerging models of animal coloration. Here, I focus on the role of the TFEC transcription factor in snake and lizard coloration based on a study on wild-type and piebald ball pythons. Genomic mapping previously identified a TFEC mutation linked to the piebald ball python phenotype. The association of TFEC with skin coloration was further supported by gene-editing experiments in the brown anole lizard. However, novel histological analyses presented here reveal discrepancies between the ball python and the anole TFEC mutants phenotype, cautioning against broad generalizations. Indeed, both wild-type and piebald ball pythons completely lack iridophores, whereas the TFEC anole lizard mutants lose their iridophores compared to the wild-type anole. Based on these findings, I discuss the potential role of the MiT/TFE family in skin pigmentation across vertebrate lineages and advocate the need for developmental analyses and additional gene-editing experiments to explore the reptilian coloration diversity.

## 1 Introduction

Although the zebrafish remains the main reference in the animal coloration field (Patterson and Parichy, 2019), reptilian species are gaining ground as new models (Milinkovitch and Tzika, 2007) thanks to the great diversity of color and color patterns they exhibit. Multiple studies have investigated the distribution of chromatophores in their skin at late stages of development or in adults to understand this colorful variety (Kuriyama et al., 2020), but little is known about the developmental processes involved in the differentiation, migration and self-organised patterning of these cells. The characterization of spontaneously-occurring mutations that affect the coloration of snakes and lizards (Andrade et al., 2019; Ullate-Agote et al., 2020; Ullate-Agote and Tzika, 2021; Garcia-Elfring et al., 2023) and the establishment of gene-editing protocols using CRISPR-Cas9 in the same lineages (Rasys et al., 2019; Tzika et al., 2023) will greatly advance our understanding of these processes, but it remains to be seen how transferrable this information is within reptiles, and Squamates (snakes and lizards) in particular.

Garcia-Elfing *et al.* (Garcia-Elfring et al., 2023) recently investigated the piebald ball python morph (*Python regius*), a recessive mutant phenotype characterized by the presence of white patches on its otherwise black and brown dorsal skin pattern. Their genome mapping analyses, and candidate-gene approach provide evidence for a mutation in the gene *TFEC* as the genetic determinant of the piebald phenotype in ball pythons. The authors also generated, through CRISPR-Cas9 gene-editing, a targeted knock-out mutation of *TFEC* in the brown anole lizard (*Anolis sergei*), resulting in the loss of iridophores and reduced body coloration, particularly in the snout, forelimbs, and hindlimbs. But as Garcia-Elfing *et al.* (Garcia-Elfring et al., 2023) conclude, it remains “unresolved whether piebald ball pythons have iridophores in either pigmented or white skin”, and they strongly recommend histological analyses to resolve this matter. Here, I present such histological data demonstrating that both wild-type and piebald ball pythons lack iridophores. Thus, the brown anole phenotype does not recapitulate the observations in ball pythons. Nevertheless, these results significantly advance our understanding of reptilian coloration. The work of Garcia-Elfing *et al.* and the results presented here suggest that the same molecule can affect reptilian and vertebrate coloration in different ways.

## 2 Materials and methods

### 2.1 Animal experimentation

Ball pythons and corn snakes were housed and bred at the LANE animal facility running under veterinary cantonal permit no. 1008. The individuals were sampled following Swiss law regulations and under the experimentation permit GE24/33145.

### 2.2 Histology and imaging

Three wild-type (histology, TEM, eye) and one piebald (histology, TEM, eye) pythons and two wild-type (histology, eye) and one Palmetto (histology) corn snakes were sampled. Skin (roughly 1 cm × 2 cm) and eye samples were fixed in 4% paraformaldehyde and dehydrated in ethanol before embedding in paraffin blocks. Seven-micrometer microtome sections were deparaffinized and directly imaged with the VHX-7000 (Keyence). For transmission electron microscopy, skin pieces of 1 mm^2^ were fixed, sectioned, and imaged as previously described (Ullate-Agote et al., 2020). Sample processing and imaging were performed at the Electron Microscopy Facility, University of Lausanne (Switzerland).

### 2.3 *TFEC* amplification

For all animals (Table 1), genomic DNA was extracted from whole blood using the DNeasy Blood & Tissue Kit (Qiagen, 51104) and genotyping by Sanger sequencing, targeting the *TFEC* SNP identified in the original publication (Garcia-Elfring et al., 2023), was performed with the following primers: TFECg_103193F (CAG​TGC​AAC​TCA​AAG​GGA​ACA) and TFECg_103880R (GCA​GAC​CCA​TGA​AAT​CAA​TGG​A).

**TABLE 1:** Individuals genotyped for the presence of the SNP suggested to cause the piebald ball python phenotype. The SNP is highlighted in bold in the ‘sequence’ column. The ‘genotype’ is deduced by the appearance and the pedigree of the animals. ‘Stripe’ is another ball python morph unrelated to the piebald morph.

Individual ID	Phenotype	Genotype	Sequence
PREG003	wild-type	+/+	CAC​AGA​TAC​A**C**GAG​CAA​TGG​C
PREG006	wild-type	piebald/+	CACAGATACA**Y**GAGCAATGGC
PREG008	piebald	piebald/piebald	CAC​AGA​TAC​A**T**GAG​CAA​TGG​C
PREG025	stripe	+/+	CAC​AGA​TAC​A**C**GAG​CAA​TGG​C
PREG037.24	wild-type	piebald/+	CACAGATACA**Y**GAGCAATGGC
PREG043	wild-type	+/+	CAC​AGA​TAC​A**C**GAG​CAA​TGG​C
PREG052	piebald	piebald/piebald	CAC​AGA​TAC​A**T**GAG​CAA​TGG​C

## 3 Results

Garcia-Elfing *et al.* (Garcia-Elfring et al., 2023) provide convincing evidence, from their genomic mapping analyses, that the mutation responsible for the piebald phenotype in ball pythons resides in an 8-Mb interval on the Chromosome 7 of the Burmese python genome. Among the variants detected within the protein-coding sequence of 32 genes in the interval, only one is expected to affect the structure of the corresponding protein. Indeed, this variant introduces a STOP codon in exon 5 of *TFEC,* and the transcription factor produced is truncated. A single copy of *TFEC* is present in reptilian genomes. Based on Sanger sequencing of exon 5 from 7 animals (3 wild-type, 2 homozygous piebald and 2 heterozygous piebald), I was able to confirm the co-segregation of the C-to-T transition in *TFEC* with the piebald allele (Table 1) in individuals that were not included in the original study.

**FIGURE 1:**
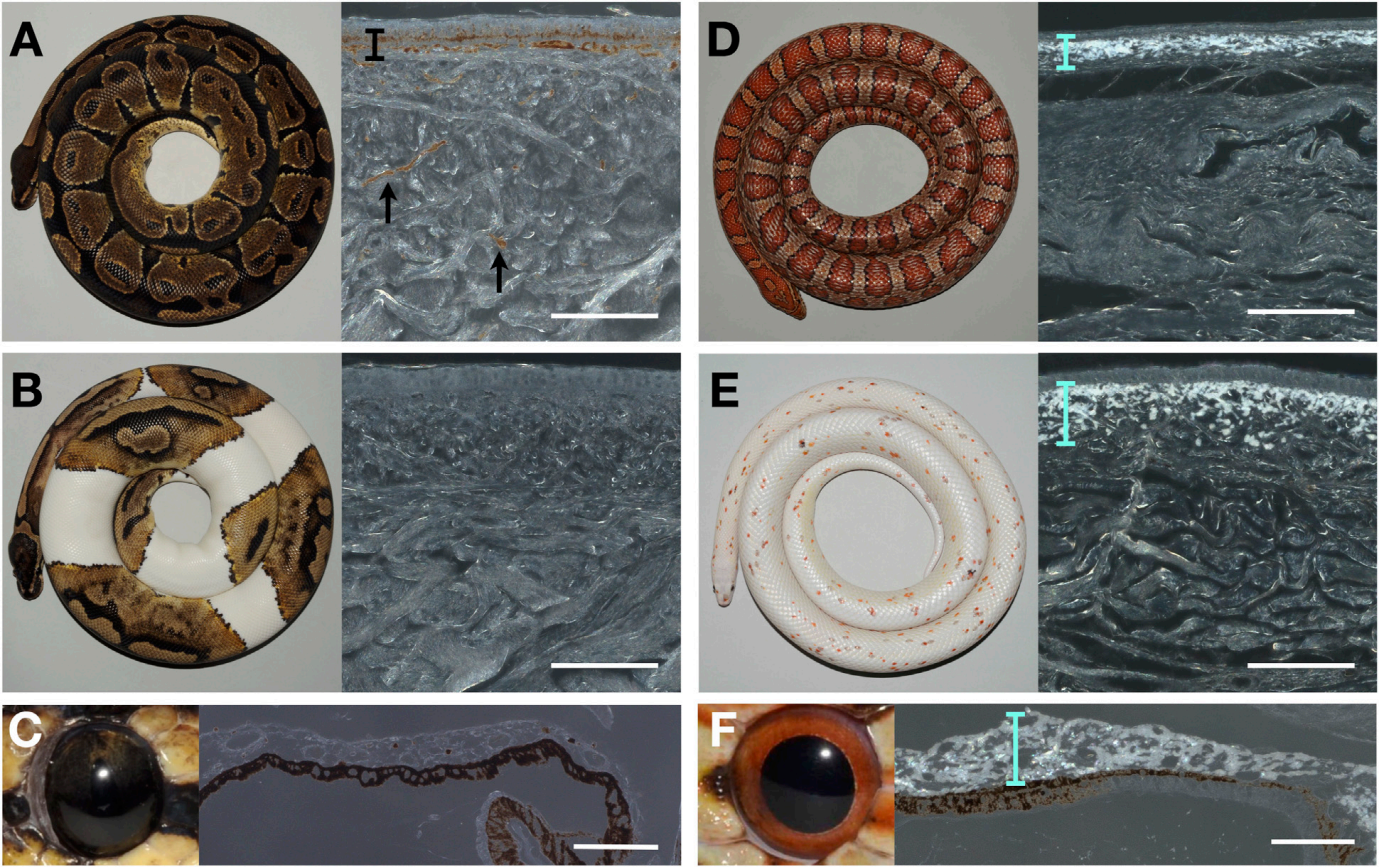
Photography of animals (left) and microscopy image of a deparaffinized dorsal skin section (right) of **(A)** a wild-type adult ball python (black skin), **(B)** a piebald ball python (white patch), **(D)** a wild-type corn snake (dorsal side), and **(E)** a Palmetto corn snake (white patch). Photography of an eye (left) and microscopy image of a deparaffinized eye section from a wild-type ball python **(C)** and a wild-type corn snake **(F)**. The black bar and arrows indicate the melanophores in **(A)**. Cyan bars highlight the layer of iridophores in D, E, and F. Scale bars: 100 μm.

I proceeded to characterize histologically the chromatophore composition of the ball python skin both in the wild-type and in piebald mutants. When coaxial light illuminates deparaffinised sections of the black skin of a wild-type ball python, the characteristic reflective shine of iridophores is absent (Figure 1A). The same result is obtained when illuminating sections of white skin patches (Figure 1B) from a piebald python. Similarly, no iridophores were detected in the iris of the wild-type ball python (Figure 1C), where iridophores have previously been reported in other reptiles, such as the Texas rat snake (Ullate-Agote and Tzika, 2021). This finding must be contrasted to the result obtained when performing the same experiment with skin and eye samples of the corn snake (*Pantherophis guttatus*): the bright reflection of light by iridophores is visible both in the wild-type skin (Figure 1C) and in the skin of the recessive Palmetto phenotype (Figure 1D), as well the eyes of the wild-type corn (Figure 1F). The Palmetto corn snake morph is characterized by fully white dorsal and ventral skin with scarce patches of coloration; the causative mutation for this phenotype remains unknown. Note that the full-depth skin pieces for paraffin sections were large, so any iridophores present could not have been missed.

**FIGURE 2:**
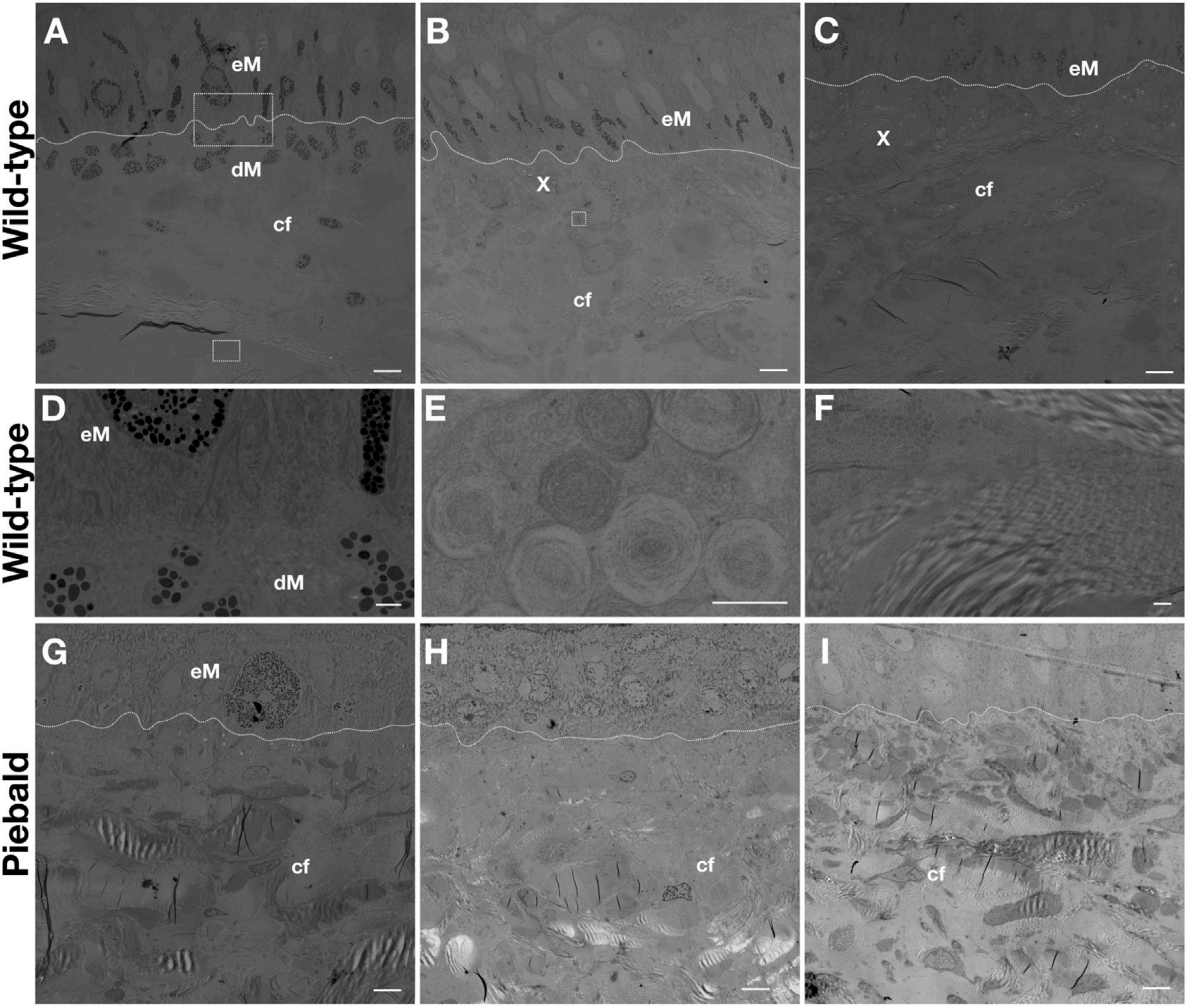
TEM imaging of the dorsal black **(A)**, dorsal brown **(B)** and lateral light brown **(C)** skin of an adult wild-type ball python. Magnification of the dermal and epidermal melanophores from A **(D)**, a xanthophore from B **(E)**, and the collagen fibers from C **(F)**. TEM imaging of the dorsal brown **(G)**, lateral white **(H)**, and ventral white **(I)** skin of a piebald adult ball python. The dashed lines mark the dermis/epidermis boundary. eM: epidermal melanophore, dM: dermal melanophore, X: xanthophore, cf: collagen fibers. Scale bars: 5 μm **(A,B,C,G,H, I)**, 1 μm **(D, F)**, 0.5 μm **(E)**.

Transmission electron microscopy (TEM) imaging of the skin of a wild-type adult ball python shows that i) epidermal and dermal melanophores are present in the dorsal black skin (Figure 2A), ii) epidermal melanophores and xanthophores can be found in the dorsal brown skin (Figure 2B), and iii) the lateral light brown skin (Figure 2C) has a similar composition but the epidermal melanophores seem more scarce. The dermal and epidermal melanophores present similar subcellular morphology (Figure 2D). The presence of concentric lamellae in the xanthosomes (Figure 2E) suggests that they contain pteridines, and they could thus be pterinosomes, but chemical analyses are necessary to confirm this. Here, I use the terms xanthophores and xanthosomes as more generic, rather than to specify the content (pteridines vs carotenoids) of these chromatophores. All pieces of skin contain large amounts of collagen fibers in various orientations (Figure 2F). The subcellular structure of the ball python melanophores and xanthophores strongly resembles that of other snakes, such as the corn snake [Figure 4 in Ullate-Agote et al. (2020)] and the Texas rat snake [Figure 2 in Ullate-Agote and Tzika (2021)].

The dorsal brown skin of a piebald individual (Figure 1G) contains epidermal melanophores and xanthophores. In the white lateral patches (Figure 1H) and the white ventral skin (Figure 1I), only dense collagen fibers can be seen. Thus, the white color we perceive in ball pythons, both on the ventral skin of wild-type animals and on the white dorso-lateral patches of piebald mutants, is likely caused by light scattering from the dense network of collagen fibers, rather than by disorganized lattices of guanine nano-crystals in iridophores. Conversely, TEM imaging of the skin on *TFEC* brown anole mutants (Garcia-Elfring et al., 2023) showed that they maintain both melanophores and xanthophores, but lose iridophores, explaining why the entire skin of these animals is translucent. Note that, although their coloration is reduced, it is unclear if the pattern itself (*i.e.*, the spatial distribution of colored motifs) is affected in these gene-edited lizards. In conclusion, the data presented here show that the phenotype of *TFEC* mutants of the brown anole does not recapitulate the effect of the *TFEC* mutation in piebald ball pythons, advocating that extrapolations of results between different species can be illuminating but must be made with caution.

## 4 Discussion

**FIGURE 3:**
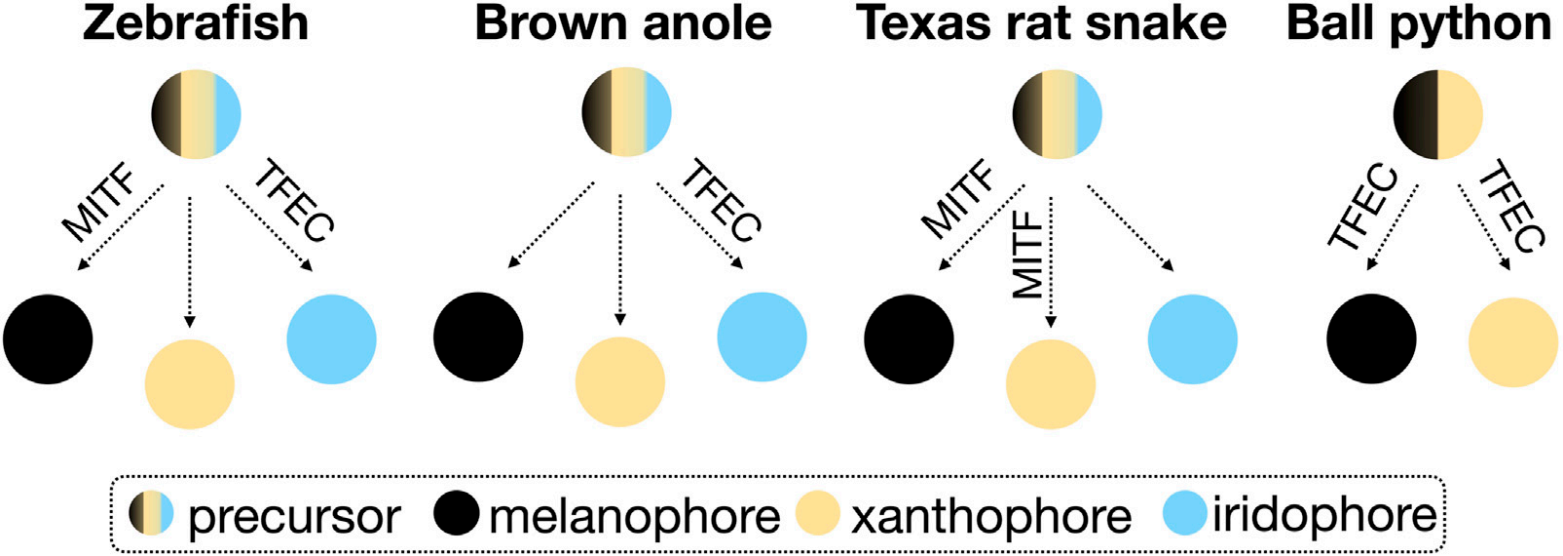
Schematic representation of the possible association of MITF and TFEC in the differentiation of the three chromatophore types from neural crest precursor cells in zebrafish, snakes and lizards. The fate determination processes are disregarded in this simplified scheme.

TFEC is part of the microphthalmia/transcription factor E (MiT/TFE) family of transcription factors, which additionally includes MITF (Melanocyte Inducing Transcription Factor), TFEB (Transcription Factor EB), and TFE3 (Transcription Factor Binding to IGHM Enhancer 3). So far, TFEB and TFE3 have not been associated with the development of animal skin coloration. In mice, a *Tfec* knockout presents abnormal hair pigmentation (Groza et al., 2023), whereas mice with a partially truncated *Tfec,* as in the piebald pythons, are normally pigmented (Steingrimsson et al., 2002). *Mitf* mutations in mice result in decreased or absent pigmentation and, occasionally, in white spotting (Steingrimsson et al., 2004). In zebrafish, *mitfa* mutants are characterized by the loss of melanophores and an increased number of iridophores (Lister et al., 1999), and *tfec* mutations mainly impact the differentiation of iridophores (Petratou et al., 2021). Reduced *MITF* expression in the Texas Rat snake results in the loss of melanophores and xanthophores (Ullate-Agote and Tzika, 2021). If we focus on the phenotype of gene knockout animals (induced or spontaneously-occurring), rather than on the proposed models describing the fate determination processes of chromatophores [progressive, direct, and cyclical (Kelsh et al., 2021)], we can speculate that the role of MITF and TFEC varies in different vertebrate lineages. Possible associations based on the scarce existing data on reptiles are presented in the simplified scheme of Figure 3. Depending on the chromatophore types present in a species, these transcription factors are likely to take up different functions in the chromatophore fate determination.

To elucidate the actual role of these transcription factors in reptilian coloration, it is thus necessary to investigate further their evolutionary history across lineages, for example, by comparing their protein structure and expression levels during development. In this study, I only sampled adult individuals, so I cannot exclude the possibility that iridophores are present in ball pythons during embryogenesis and disappear as the animals grow. Indeed, there are reptiles, like the ocellated and other lizards (Fofonjka and Milinkovitch, 2021; Jahanbakhsh and Milinkovitch, 2022), whose skin coloration and pattern changes continuously. This is not the case though for ball pythons; they maintain the same skin coloration and pattern throughout their lives. Nevertheless, it is necessary to investigate the differentiation of their chromatophores during development. Transcriptomic analyses can help us identify gene markers of reptilian chromatophores, such that we track them during embryogenesis. Undoubtedly, gene-editing experiments in multiple species, as performed in the brown anole (Garcia-Elfring et al., 2023), would also illuminate how differentiation, maturation, migration, and survival of different chromatophores—as well as their interactions—have evolved to produce the remarkable diversity of colors and patterns in Squamate reptiles.

## Data Availability

The original contributions presented in the study are included in the article, further inquiries can be directed to the corresponding author.
